# A Clinic on a Fusible Alloy

**Published:** 1898-04

**Authors:** Grant Molyneaux


### A Clinic on a Fusible Alloy.

BY GRANT MOLYNEAUX, D.D.S.

The increased number and great variety of operations performed in the modern dental office, entailing as they do, a considerable amount of mechanism, compell the busy practitioner to seek the most rapid method for the execution of his work. The construction of splints, supports for loose teeth under treatment, regulating and retaining appliances, " bridges," crowns, etc., can only be properly executed under the direct supervision of the attending deniist. If he be a busy man, both time and patience are taxed to the utmost by the cumbersome and clumsy methods generally employed in the execution of the purely mechanical details. The methods generally employed, are to procure an impression either in modeling compound or plaster of paris, followed by the mixing and pouring of a plaster model, varnishing, waxing, sand-molding for die, counter-die and swaging. This requires two or more visits of the patient when one visit should suffice.

Rapidity in dental operations is always sought, but any " new method " claiming such virtues, is generally approached with fear and trembling, especially by our older brother. To allay that fear I will state that the subject of this clinic has been known to the dental profession for a quarter of a century, and as possessing many virtues, the greatest of which, has been overlooked. In advocating this new (?) rapid method, we fully appreciate that old adage, "make haste slowly," for rapid methods are only valuable when they are *accurate* and *perfectly understood* by the operator.

Wood's metal has been used in dentistry for many years and is known to contain bismuth, cadmium, tin and lead. The alloy before you contains the same ingredients as Wood's metal, the proportions being changed so as to produce an alloy that may be cast into a modeling compound or wet plaster of paris impression and give a smooth accurate model or die in metal. The advantage of being able to cast metal directly with wet plaster or modeling compound, can be appreciated by all practitioners of experience, if the models or die be accurate.

We feel safe in saying that a metal composed of five (5) parts of bismuth, three (3) parts of lead, two (2) parts of tin and two (2) parts of cadmium, properly compounded will produce when poured into either of the above-named impression materials, a more perfect model than can be obtained by the use of plaster. But a model of this metal can not be used in place of plaster in all cases, as in vulcanite, or celluloid work, for the fusing point of the metal is about 130° F.

It is especially designed for the making of a perfect die and counter-die with the expenditure of not over five minute's time, and with the very simplest kind of apparatus. By the use of such an alloy the difficulties of sand-molding are overcome and the production of a perfectly adapted plate is the result. To successfully use this, or any low-fusing alloy, several points must be constantly observed: 1st. Castings are sharpest and nearest perfect when the metal is poured close to the congealing point. 2d. Overheating causes a loss of time and deterioration of the alloy. 3d. To make a perfect and smooth casting in modeling compound the impression should be first oiled and then the alloy is cast in a mush-like consistency, when it will fall in a thick soft mass into the impression which is quickly jarred on the table, cooled in water and separated. A little practice will enable the operator to produce a perfect model in every instance. 4th. Take a plaster impression directly from the mouth, soak it thoroughly with sperm oil, and pour the alloy at a little higher temperature than for modeling compound and let it stand until cold. 5th. In order to obtain a thick base for the model take a thin copper strip, in lieu of this take a strip of heavy writing paper, about ten or twelve inches long and two inches wide, wrap around the impression and hold in place by snapping over it a small rubber band. Fill in spaces between band and impression with soft putty which will always be ready for use by being kept under water. A half-pound screw-top vaseline jar half filled with soft putty and covered with water will keep pretty soft for years. 6th. To make counter-die, wrap the copper strip around the base of the die and fill all undercuts and unnecessary parts with the putty, paint over the surface with whiting dissolved in water or alcohol, and cast the alloy as cold as possible.

Before remelting castings they should be cleaned of all putty and other dirt; if, however, the metal becomes contaminated, it can be heated until it becomes perfectly fluid, when the impurities can be removed with a piece of blotting paper. One illustration of the use of this alloy may be suggestive of its many valuable applications: In adapting a gold or platinum base for full dentures where the recession over the tuberosities and anterior ridge is so great as to make sand-molding without a core absolutely impossible. Make the model or die of fusible alloy by casting into the impression. For such a case always use plaster, as this can be broken off in such a manner as to be restored and a second die cast. Upon this second, the relief or vacuum chamber, made of block tin can be attached, with thick shellac varnish, or the relief can be first trimmed out of the impression. Use the second die for the first stamping of the plate, making the adaptation to the undercut as close as possible with riveting hammer. Try the plate in the mouth and properly trim and wire if necessary. Replace on the used die and wrap the plate and die with one covering of cheese-cloth or thin paper, and place in the Parker shot swedging device and swedge. The plate can not now be removed from the die, but by placing the same in hot water the metal will run out of the plate, leaving it unchanged in shape. It can now be polished, and after transferring the relief from the old die to the unused one, the plate is sprung onto it, and swaging with shot and melting the metal out as before, will leave the plate with an adaptation that can not be procured by any other method,

In taking an impression for metal castings, it should be a little thicker than usual, and any number of dies can be made from the same impression, all of which will be alike.

The compounding of this alloy requires the greatest of care in protecting it from the action of the air, during the first melting and in the manner of adding the metals, as it never again approaches the first heat, except by carelessness, the metal will remain permanent in composition and working qualities indefinitely.

The necessary expense of this alloy, which at first may seem unreasonable, will be saved in the saving of time in one difficult case. After two years of constant use of this metal I can positively state that it will meet all the claims of this clinic.

